# AS YOU SOW, SO SHALL YOU REAP: ADULTS' MEMORIES' EFFECT OF PARENTAL ACCEPTANCE REJECTION IN CHILDHOOD ON AGING PARENTS

**DOI:** 10.1093/geroni/igac059.150

**Published:** 2022-12-20

**Authors:** Sumbleen Ali, Ronald Rohner

**Affiliations:** University of Connecticut, Storrs, Connecticut, United States; University of Connecticut, Storrs, Connecticut, United States

## Abstract

Little is known how adults’ memories of parental acceptance-rejection in childhood influence their behavior toward their aging parents. Grounded in interpersonal acceptance-rejection theory (IPARTheory), this study attempts to better understand how early parent-child relationships affect adult offspring who provide care to their parents in later life. Data were collected from 41 adult offspring. Findings revealed that adults who felt rejected by their parents in childhood reported fewer positive caregiving behaviors toward their now aging parents, were less satisfied with social activities with their parents, spent less time with them or visited them less frequently, and revealed less overall concern for their aging parents. Results were consistent with IPARTheory’s expectations that if parents reject their children, then parents place their own dependent old age at the risk of counter rejection: As you sow, so shall you reap. Such findings may help researchers, clinicians, and practitioners better understand the well-being of aging adults.

